# Laparoscopic Pancreatoduodenectomy: Twenty years later, where are we?

**DOI:** 10.1590/0100-6991e-20243753-en

**Published:** 2024-06-14

**Authors:** ENIO CAMPOS AMICO, JOSÉ JUKEMURA

**Affiliations:** 1 - Universidade Federal do Rio Grande do Norte, Departamento de Medicina Integrada - Natal - RN - Brasil; 2 - Faculdade de Medicina da Universidade de São Paulo, Disciplina de Cirurgia do Aparelho Digestivo - São Paulo - SP - Brasil

**Keywords:** Pancreaticoduodenectomy, Laparoscopy, Pancreatic Neoplasms, Postoperative Complications, Pancreaticoduodenectomia, Laparoscopia, Neoplasias Pancreáticas, Complicações Pós-operatórias

## Abstract

In its 20^th^ anniversary, laparoscopic pancreatoduodenectomy, while feasible and safe in the hands of experienced surgeons, has not seen the anticipated popularity observed in other digestive surgery procedures. The primary hurdle remains the absence of a clear advantage over traditional open surgery, paired with the procedures complexity and a consequent steep learning curve. In regions with limited pancreatic surgery services, conducting this procedure without adequate training can have serious repercussions. Given the advent of robotic platforms and the anticipation of prospective and randomized studies on this new technology, it is imperative to engage in comprehensive discussions, endorsed by surgical societies, on the value, application, and implementation strategies for various minimally invasive pancreatoduodenectomy techniques. Such dialogue is crucial for advancing the field and ensuring optimal patient outcomes.

## RESUMO

No ano em que completa 20 anos, a Pancreatoduodenectomia Laparoscópica, embora factível e segura em mãos experientes, não obteve uma esperada popularização como aquela observada em vários outros procedimentos no campo da cirurgia digestiva. A ausência de uma clara vantagem sobre a cirurgia aberta associada a complexidade do procedimento com consequente difícil e demorada curvas de aprendizado são os principais obstáculos. Em nosso país onde se dispõe de poucos serviços de cirurgia pancreática as consequências da prática desse procedimento sem um adequado treinamento pode trazer sérios prejuizos. Torna-se imperativo, agora com difusão das plataformas robóticas e enquanto se aguarda os estudos prospectivos e randomizados com essa nova tecnologia, que uma ampla discussão seja feita com chancela das sociedades de cirurgia sobre o valor, a aplicação e a forma de implantação das diversas técnicas de pancreatoduodenectomia minimamente invasivas.

The benefit that minimally invasive procedures have provided over the last 30 years in various areas of abdominal surgery is undeniable, including in the field of oncology. Procedures that in the past caused pain, a higher degree of general complications, as well as a long recovery are now simpler, with a lower degree of discomfort and suffering to patients. It is for this reason, with the intention of extending this benefit to pancreatic surgery, that laparoscopic pancreatoduodenectomy (LPD) has, especially in the last decade, gained popularity. However, unlike other scenarios and despite a large volume of publications, the advantages of this procedure remain uncertain and most of the time they have been considered only “non-inferior” in comparison with open pancreatoduodenectomy (OPD)^1^. Recently, an interesting study published in the journal “Surgery” and carried out in a prestigious North American center, the Mayo Clinic in Jacksonville, Florida reignited the discussion. In the study, Stauffer et al.^2^ performed a comparative analysis with propensity score matching 187 patients undergoing LPD with 187 individuals submitted to OPD between 2010 and 2020 at the institution^2^. Regarding intraoperative data, the overall blood loss was statistically lower with LPD, although surgery time was statistically longer. There was no statistical difference between the length of hospital stay in the two groups (six days for OPD vs. seven for LPD, p=0.31). As for postoperative complications, although the rate of major complications (Clavien-Dindo ≥3) was statistically similar between the two procedures (21.5% in OPD vs. 27.4% in LPD), several parameters were superior with open surgery: lower clinically relevant pancreatic fistula index (5.4 vs. 18.8%), lower rate of delayed gastric emptying (9.7% vs. 18.8%), less postpancreatectomy hemorrhage (4.8% vs. 9.7%), fewer intraperitoneal abscesses (12.9% vs. 19.9%), less need for postoperative imaging (34.9% vs. 45.2%), less need for percutaneous drainage (12.9% vs. 21.0%), and lower 90-day mortality (2.2% vs. 4.3%), although only the first two parameters displayed statistical significant differences. Finally, as a logical consequence, LPD was statistically more expensive than OPD (US$ 68,479 vs. US$ 58,804). From these results, the authors claimed to have experienced in the last three years of the study a retrograde movement back from LPD to OPD. More than that, the authors go so far as to suggest that it is unlikely that robotic access can supplant the results obtained with OPD in their center, although they still consider this statement to be premature.

More than just comparing the results between LPD and OPD, the data from the study by Stauffer et al. reveal a trend that has been overshadowed by the spotlight of minimally invasive surgery: the popularization of strategies to accelerate postoperative recovery and the encouragement of early removal of abdominal drains in the postoperative period of pancreatoduodenectomy (PD) have improved results in the short term and also reduced the length of hospital stay also in the open surgery group. This is particularly true for most patients who end up having no postoperative complications. In the literature, it is increasingly common to find, in the experience of large centers that continue to practice OPD, an average hospital stay of no more than eight days. Bassi et al.^3^ retrospectively analyzed 3,000 consecutive OPDs at the Verona Pâncreas Institute in Italy and found a mean length of stay of eight days in the population of 80% of patients who did not develop major complications (Clavien-Dindo ≥3). A recent systematic review that included 31 studies with 5,382 patients undergoing PD and stratified between 2,776 patients who underwent a recovery acceleration protocol and 2,606 patients who followed conventional postoperative care found a reduction of 3.15 days in the length of hospital stay in the first group, a result consistent with others with the same methodology^4^. The most impressive result was recently (2023) published by Ayabe et al.^5^, updating the institutional care protocol for patients undergoing PD at the MD Anderson Cancer Center with intensive postoperative follow-up support. In 80% of the cases, classic OPD without pylorus preservation and manual gastrojejunostomy was the standard procedure. Even with a readmission rate considered high (29%), the authors observed a mean length of stay of four days in the group of patients at low risk of developing pancreatic fistula. In that same group, the rate of major complications was only 14%. 

The Jacksonville Mayo Clinic study isn’t the first to question the value of LPD. This has occurred with other publications, including those showing LPD benefits. Wang et al. published the largest randomized study to date, which included 14 Chinese medical centers with a high volume of pancreatic surgery^6^. To enroll in the study, a personal experience of 104 open or laparoscopic procedures was required for each surgeon included, a very high value compared with other studies with the same methodological design. Despite the shorter length of hospital stay with LPD and some other better minor parameters, due to similar morbidity and mortality results the authors concluded that the benefits of the laparoscopic technique are marginal and that future studies are needed to define which population can effectively benefit from the procedure.

The absence of clear benefit from LPD in our midst is unfortunately only one side of the coin. In general, innovation in the field of pancreatic surgery faces another important challenge: the small number of pancreatic surgery centers available that perform an adequate number of pancreatectomies per year and thus fit the definition of “high volume” hospitals. According to the recent Brescia Guideline, an update of the 2019 Miami Guideline, morbidity, mortality, and R0 resection rate for cancer are better when PD is performed in centers with an annual volume of 20 or more cases^7^. In Brazil, at least in public institutions, this number is difficult to achieve. Szor et al. investigated PDs performed by institutions of the Brazilian Public Health System between 2008 and 2021, using a value of eight or more procedures per year to define a hospital as “high volume”, and found that only 10 hospitals out of 283 (3.5%) fit this definition^8^. For this “high volume” group, in-hospital mortality, although high, was statistically lower then “low volume” hospitals (8% vs. 17%). Considering that the implementation of LPD involves a selection of simpler cases a priori and that the centers are composed of more than one surgeon, it is foreseeable that even in these centers considered “high volume”, several years will be necessary for a few surgeons to reach the long learning curves with the complex PD procedure. 

In recent years, with the popularization of robotic surgery platforms in the world, there has been a natural migration from laparoscopic to robotic access in pancreatic head resection procedures. It seems reasonable that, due to its better ergonomics for the surgeon, especially in the reconstruction phase, robotic pancreatoduodenectomy (RPD) will in fact improve the results of LPD, even expanding the number of surgeons able to perform the procedure^9^. Several case series, non-randomized comparative studies, and some reviews have shown that in experienced hands, the procedure is feasible, safe, and with immediate benefits, such as less blood loss and shorter hospital stay, among others^10^. The results of randomized trials are still expected and should ideally be performed by institutions with expertise in both robotic and open surgery, with a focus on defining the superiority of one over the other in the various patient populations and specific diseases. A safe way to implement minimally invasive PD techniques should also be considered, preferably under the supervision of specialized surgical societies, considering the human and economic impacts of this implementation.

Since the first laparoscopic procedures were performed, PD has been one of the last frontiers of minimally invasive surgery and there is no doubt that it already occupies a well-deserved space in the wide range of treatment possibilities for pancreatic diseases. The issue we consider pertinent is that, unlike several other less complex procedures in the field of digestive surgery, its implementation has important and essential steps: 1) before starting minimally invasive PD training, a solid experience in OPD and also in general minimally invasive surgery is required; 2) training should preferably be carried out in centers with a significant volume of annual procedures and in accordance with international guidelines; 3) it is essential that cases with a lower degree of complexity are initially chosen; 4) results should be carefully evaluated and compared with those obtained in the conventional procedure; and 5) one should recognize that only a small part of digestive surgeons will be able to achieve these steps, finally achieving the appropriate proficiency to perform this complex procedure.

## References

[1] Yin T, Qin T, Wei K, Shen M, Zhang Z, Wen J (2022). Comparison of safety and effectiveness between laparoscopic and open pancreatoduodenectomy A systematic review and meta-analysis. Int J Surg.

[2] Stauffer JA, Hyman D, Porrazzo G, Tice M, Li Z, Almerey T (2024). A propensity score-matched analysis of laparoscopic versus open pancreaticoduodenectomy Is there value to a laparoscopic approach?. Surgery.

[3] Bassi C, Marchegiani G, Giuliani T, Di Gioia A, Andrianello S, Zingaretti CC (2022). Pancreatoduodenectomy at the Verona Pancreas Institute the Evolution of Indications, Surgical Techniques, and Outcomes: A Retrospective Analysis of 3000 Consecutive Cases. Ann Surg.

[4] Noba L, Rodgers S, Doi L, Chandler C, Hariharan D, Yip V (2023). Costs and clinical benefits of enhanced recovery after surgery (ERAS) in pancreaticoduodenectomy an updated systematic review and meta-analysis. J Cancer Res Clin Oncol.

[5] Ayabe RI, Prakash LR, Bruno ML, Newhook TE, Maxwell JE, Arvide EM (2023). Differential Gains in Surgical Outcomes for High-Risk vs Low-Risk Pancreaticoduodenectomy with Successive Refinements of Risk-Stratified Care Pathways. J Am Coll Surg.

[6] Wang M, Li D, Chen R, Huang X, Li J, Liu Y (2021). Minimally Invasive Treatment Group in the Pancreatic Disease Branch of China's International Exchange and Promotion Association for Medicine and Healthcare (MITG-P-CPAM) Laparoscopic versus open pancreatoduodenectomy for pancreatic or periampullary tumours: a multicentre, open-label, randomised controlled trial. Lancet Gastroenterol Hepatol.

[7] Abu Hilal M, van Ramshorst TME, Boggi U, Dokmak S, Edwin B, Keck T (2024). The Brescia Internationally Validated European Guidelines on Minimally Invasive Pancreatic Surgery (EGUMIPS). Ann Surg.

[8] Szor DJ, Tustumi F (2023). The influence of institutional pancreaticoduodenectomy volume on short-term outcomes in the Brazilian public health system 2008-2021. Rev Col Bras Cir.

[9] Machado MAC, Makdissi FF (2021). ASO Author Reflections The Role of the Robot in Pancreatoduodenectomy. Ann Surg Oncol.

[10] Mantzavinou A, Uppara M, Chan J, Patel B (2022). Robotic versus open pancreaticoduodenectomy, comparing therapeutic indexes; a systematic review. Int J Surg.

