# Erratum

**DOI:** 10.1590/0100-6991e-20243753errata-en

**Published:** 2025-01-31

The article published under the DOI number 10.1590/0100-6991e-20243753-en, published in the Journal of the Brazilian College of Surgeons. 51:e20243753.

In the title, where it is:

“Laparoscopic Pancreatoduodenectomy: Twenty years later, where are we?”

It should be:

“Laparoscopic Pancreatoduodenectomy: Thirty years later, where are we?”

And where it is:

“ABSTRACT: In its 20th anniversary, laparoscopic pancreatoduodenectomy, while feasible and safe in the hands of experienced surgeons, has not seen the anticipated popularity observed in other digestive surgery procedures. (…)”

It should be:

“ABSTRACT: In its 30th anniversary, laparoscopic pancreatoduodenectomy, while feasible and safe in the hands of experienced surgeons, has not seen the anticipated popularity observed in other digestive surgery procedures. (…)”

